# Preoperative cardiac troponin below the 99th-percentile upper reference limit and 30-day mortality after noncardiac surgery

**DOI:** 10.1038/s41598-020-72853-3

**Published:** 2020-10-12

**Authors:** Jungchan Park, Cheol Won Hyeon, Seung-Hwa Lee, Sangmin Maria Lee, Junghyun Yeo, Kwangmo Yang, Jeong Jin Min, Jong Hwan Lee, Jeong Hoon Yang, Young Bin Song, Joo-Yong Hahn, Seung-Hyuk Choi, Jin-Ho Choi, Hyeon-Cheol Gwon

**Affiliations:** 1 Department of Anesthesiology and Pain Medicine, Samsung Medical Center, Sungkyunkwan University School of Medicine, Seoul, Korea; 2 Division of Cardiology, Department of Medicine, Heart Vascular Stroke Institute, Samsung Medical Center, Sungkyunkwan University School of Medicine, 81 Irwon-ro, Gangnam-gu, Seoul, Korea; 3 Centers for Health Promotion, Samsung Medical Center, Sungkyunkwan University School of Medicine, Seoul, Korea; 4 Department of Emergency Medicine, Samsung Medical Center, Sungkyunkwan University School of Medicine, Seoul, Korea

**Keywords:** Biomarkers, Cardiology, Medical research

## Abstract

Preoperative high-sensitivity cardiac troponin (hs-cTn) above the 99th-percentile upper reference limit (URL) is associated with mortality after noncardiac surgery. This study aimed to evaluate whether preoperative hs-cTn concentrations above the lowest limit of detection (LOD) but below the 99th-percentile URL can predict mortality after noncardiac surgery.From January 2010 to April 2019, a total of 12,415 noncardiac surgical patients with preoperative hs-cTn I below the 99th-percentile URL were enrolled. The patients were divided into two groups according to preoperative hs-cTn I concentration: (1) [hs-cTn] below the LOD (6 ng/L), and (2) mildly elevated [hs-cTn] but below the 99th-percentile URL (40 ng/L). The primary outcome was 30-day mortality. Of the 12,415 patients enrolled, 7958 (64.1%) were in the LOD group whereas 4457 (35.9%) were in the mild elevation group. The incidence of 30-day mortality was significantly greater in the mild elevation group (2.1% vs. 4.0% hazard ratio [HR] 1.73; 95% confidence interval [CI] 1.39–2.16; p < 0.001) in the multivariate analyses. The propensity score matched analyses also produced a similar result (2.6% vs. 4.2% HR 1.61; 95% CI 1.26–2.07; p < 0.001). The threshold at which the risk of mortality increased corresponded to a preoperative hs-cTn I ≥ 12 ng/L. Patients with preoperative hs-cTn I above the LOD and below the 99th-percentile URL had greater 30-day mortality after noncardiac surgery.

## Introduction

Perioperative serum cardiac troponin concentration has recently been accepted as a strong predictor of 30-day mortality after noncardiac surgery^1,2^. Based on robust clinical relevance, the diagnostic criteria for postoperative myocardial injury after noncardiac surgery (MINS) include the occurrence of postoperative troponin concentrations above the 99th-percentile upper reference limit (URL) within 30 days post-surgery, resulting from myocardial ischemia without the requirement of an ischemic feature^3–5^. Since the establishment of the MINS criteria, subsequent studies have mainly focused on postoperative troponin concentrations and used the 99th-percentile URL to reveal relevant risk factors or optimal treatments for MINS^4,6,7^.

Preoperative cardiac troponin concentrations have also been shown to be associated with postoperative outcomes^8–11^, but this phenomenon has garnered little attention, because it precedes an enormous metabolic stress from surgical manipulations. Although baseline measurements of some cardiac biomarkers that fall within normal values have shown prognostic value in asymptomatic patients^12–14^, the presence of preoperative troponin concentrations below the 99th-percentile URL has typically been considered normal^5,15,16^. However, as highlighted by the Fourth Universal Definition, perioperative myocardial injury shows distinct features that differentiate it from myocardial infarction^5^, and several factors other than heart injury are also related to the serum troponin concentration to varying degrees^17^. Given the 99th-percentile URL provided by the high-sensitivity cardiac troponin (hs-cTn) immunoassay manufacturer was established using data from healthy individuals, this URL may not be an optimal cut-off value for patients scheduled for surgery. In addition, the association between preoperative cardiac troponin concentrations below the 99th-percentile URL and postoperative outcome has not been fully evaluated. Therefore, we aimed to evaluate the prognostic impact of preoperative serum cardiac troponin concentrations above the lowest limit of detection (LOD) but below the 99th-percentile URL.

## Results

### Baseline characteristics

The 12,415 patients were divided into the two groups according to preoperative hs-cTn I concentration: 7958 (64.1%) patients were in the LOD group and 4457 (35.9%) patients were in the mild elevation group. The patient characteristics at baseline are summarized in Table 1. The mild elevation group tended to be older and showed higher incidences of past medical events and medication use. A total of 3869 pairs of data were generated by 1:1 individual matching without replacement using the propensity scores for preoperative variables. After propensity score matching, all preoperative variables were well balanced, and we found no significant difference between intraoperative variables (Table 1).

**Table 1:** Baseline characteristics.

	Entire population				Propensity score matched population		
	LOD (N = 7958)	Mild elevation (N = 4457)	p-Value	SMD	LOD (N = 3869)	Mild elevation (N = 3869)	SMD
^a^Preoperative hs-cTn I, ng/L	6	16 (± 9)			6	16 (± 9)	
**Preoperative variables**							
Male	4432 (55.7)	2603 (58.4)	0.004	5.5	2217 (57.3)	2233 (57.7)	0.8
Age	58.0 (± 18.0)	63.9 (± 17.0)	< 0.001	34.2	62.6 (± 16.0)	63.0 (± 17.4)	2.2
Hypertension	4417 (55.5)	3322 (74.5)	< 0.001	40.7	2722 (70.4)	2741 (70.8)	1.1
Diabetes	1924 (24.2)	1528 (34.3)	< 0.001	22.4	1196 (30.9)	1190 (30.8)	0.3
Coronary artery disease	802 (10.1)	811 (18.2)	< 0.001	23.5	585 (15.1)	578 (14.9)	0.5
Chronic kidney disease	268 (3.4)	630 (14.1)	< 0.001	38.8	264 (6.8)	264 (6.8)	< 0.1
Previous stroke	485 (6.1)	397 (8.9)	< 0.001	10.7	321 (8.3)	313 (8.1)	0.8
Arrhythmia	339 (4.3)	452 (10.1)	< 0.001	22.9	287 (7.4)	303 (7.8)	1.6
Current smoking	663 (8.3)	310 (7.0)	0.01	5.2	271 (7.0)	284 (7.3)	1.3
Preoperative hemoglobin, g/dl	12.5 (± 2.0)	11.7 (± 2.2)	< 0.001	37.8	11.9 (± 2.1)	11.9 (± 2.1)	0.5
Medication							
Antiplatelet agent	2232 (28.0)	1921 (43.1)	< 0.001	31.8	1453 (37.6)	1474 (38.1)	1.1
Statin	2165 (27.2)	1637 (36.7)	< 0.001	20.5	1263 (32.6)	1277 (33.0)	0.8
Beta-blocker	1689 (21.2)	1710 (38.4)	< 0.001	38.2	1262 (32.6)	1266 (32.7)	0.2
Calcium channel blocker	2586 (32.5)	2180 (48.9)	< 0.001	33.9	1725 (44.6)	1703 (44.0)	1.1
RAAS inhibitor	2236 (28.1)	2001 (44.9)	< 0.001	35.4	1520 (39.3)	1520 (39.3)	< 0.1
ESC/ESA surgical risk			< 0.001	13			7.3
Mild	2441 (30.7)	1154 (25.9)			1112 (28.7)	1042 (26.9)	
Intermediate	4554 (57.2)	2833 (63.6)			2265 (58.5)	2397 (62.0)	
High	963 (12.1)	470 (10.5)			492 (12.7)	430 (11.1)	
Operation type			< 0.001	24.9			10.4
Vascular	982 (12.3)	672 (15.1)			489 (12.6)	507 (13.1)	
Orthopedics	2371 (29.8)	1378 (30.9)			1198(31.0)	1192 (30.8)	
Abdominal	984 (12.4)	706 (15.8)			590 (15.2)	596 (15.4)	
Thoracic	22 (0.3)	8 (0.2)			7 (0.2)	8 (0.2)	
Neuro	2437 (30.6)	911 (20.4)			1007 (26.0)	876 (22.6)	
Otolaryngology, eye	707 (8.9)	486 (10.9)			341 (8.8)	429 (11.1)	
Urology, gynecology	455 (5.7)	296 (6.6)			237 (6.1)	261 (6.7)	
Emergency	1706 (21.4)	1194 (26.8)	< 0.001	12.5	1004 (25.9)	1012 (26.2)	0.5
General anesthesia	6449 (81.0)	3183 (71.4)	< 0.001	22.7	2902 (75.0)	2905 (75.1)	0.2
^**a**^**Intraoperative variables**							
Operation duration, minute	161.0 (± 142.1)	139.9 (± 129.3)	< 0.001		157.0 (± 143.6)	145.2 (± 132.6)	8.5
Inotropic drug requirement	2798 (35.2)	1751 (39.3)	< 0.001		1487 (38.4)	1511 (39.1)	1.3
Red blood cell transfusion	2978 (37.2)	1506 (33.8)	< 0.001		1374 (35.5)	1369 (35.4)	0.3

Data are presented as n (%) or mean (± standard deviation).

*LOD* limit of detection; *hs-cTn* high-sensitivity cardiac troponin; *RAAS* renin–angiotensin–aldosterone system; *ESC* European Society of Cardiology; *ESA* European Society of Anaesthesiology.

^a^Preoperative hs-cTn level and intraoperative variables were not included in propensity score matching.

### Clinical outcomes

**Figure 1:**
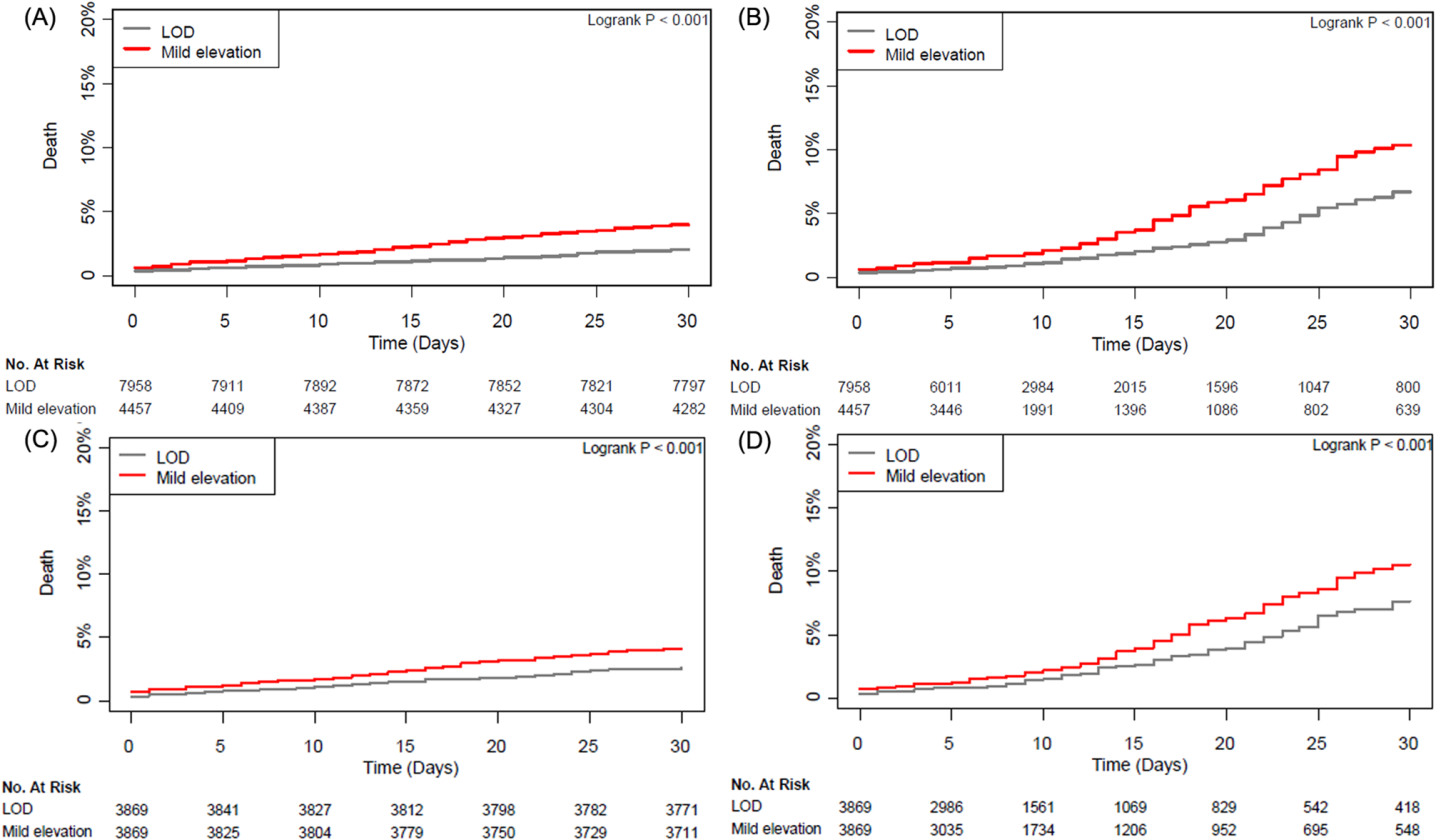
Kaplan–Meier curves for (**A**) 30-day and (**B**) in-hospital mortalities of the minimum and minor elevation groups within the full study population, and Kaplan–Meier curves for (**C**) 30-day and (**D**) in-hospital mortalities of the minimum and minor elevation groups in the propensity-score-matched population.

As we used mortality data validated from the National Population Registry of the Korea National Statistical Office, all study patients completed follow-up period of postoperative 30-day mortality. For in-hospital mortality, the median period was 8 days (interquartile range [IQR] 5–16). In the entire population, multivariate analyses showed significantly higher risks of 30-day and in-hospital mortalities for the mild elevation group (2.1% vs. 4.0%; hazard ratio [HR] 1.73; 95% confidence interval [CI] 1.39–2.16; p < 0.001 and 2.8% vs. 5.5%; HR 1.40; 95% CI 1.16–1.69; p = 0.001, respectively; Table 2). The propensity-score-matched analysis produced similar results (2.6% vs. 4.2%; HR 1.61; 95% CI 1.26–2.07; p < 0.001 and 3.6% vs. 5.7%; HR 1.38; 95% CI 1.12–1.71; p = 0.003, respectively) (Table 2). Survival curves for the entire and propensity-score-matched populations are shown in Fig. 1. Sensitivity of the effect of an unmeasured confounder on this association was calculated with an assumed prevalence of 40% for unmeasured confounder, and the association was consistently significant (Supplementary Table S1). The power of this study regarding the sample size was 0.97. In this study, the significant attributable fractions of the risk for 30-day mortality included emergency surgery (40.9%), preoperative hs-cTn I above the lowest LOD (21.2%), and high surgical risk (10.6%) (Supplementary Table S2).

**Table 2:** Clinical outcomes.

	Univariate analysis		Multivariate analysis				Propensity score matched analysis			
	LOD (N = 7958)	Mild elevation (N = 4457)	Unadjusted HR (95% CI)	p-Value	^a^Adjusted HR (95% CI)	p-Value	LOD (N = 3869)	Mild elevation (N = 3869)	Adjusted HR (95% CI)	p-Value
30-day mortality	164 (2.1)	178 (4.0)	1.96 (1.58–2.42)	< 0.001	1.73 (1.39–2.16)	< 0.001	101 (2.6)	161 (4.2)	1.61 (1.26–2.07)	< 0.001
In-hospital mortality	220 (2.8)	246 (5.5)	1.59 (1.33–1.91)	< 0.001	1.40 (1.16–1.69)	0.001	138 (3.6)	221 (5.7)	1.38 (1.12–1.71)	0.003
30-day peak hs-cTn I level, ng/L	242 (± 2862)	1179 (± 14,520)				< 0.001	160 (± 2579)	688 (± 11,558)		0.01
MINS	644 (8.1)	840 (18.8)				< 0.001	385 (10.0)	684 (17.7)		< 0.001

Data are presented as n (%) or mean (± standard deviation).

30-day peak hs-cTn I was available in 4086/7958 (51.3%) in LOD and 2529/4457 (56.7%) in mild elevation groups of the entire population.

30-day peak hs-cTn I was available in 2142/3869 (55.4%) in LOD and 2211/3869 (57.1%) in mild elevation groups of the propensity score matched population.

Patients without elevated postoperative hs-cTn I were regarded as not having been diagnosed with MINS.

*LOD* limit of detection; *HR* hazard ration; *CI* confidential interval; *hs-cTn* high-sensitivity cardiac troponin; *MINS* myocardial injury after noncardiac surgery.

^a^Covariates include age, hypertension, diabetes, coronary artery disease, chronic kidney disease, previous stroke, arrhythmia, preoperative hemoglobin, antiplatelet agent, statin, beta-blocker, calcium channel blocker, renin–angiotensin–aldosterone system inhibitor, surgical risk, operation duration, and intraoperative inotropic use and red cell transfusion.

**Figure 2:**
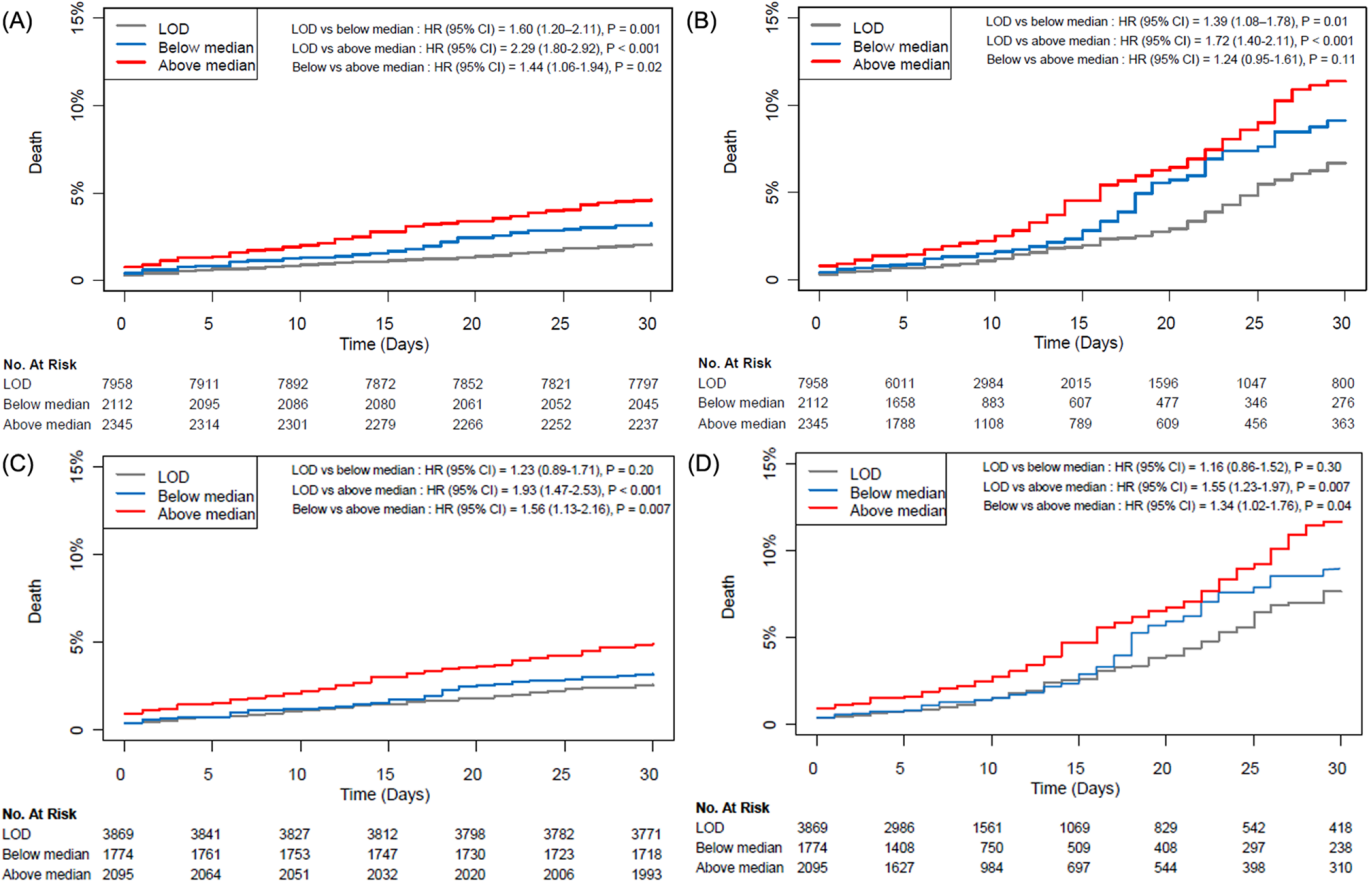
Kaplan–Meier curves of three groups according to the median preoperative high-sensitivity cardiac troponin I concentrations; (**A**) 30-day mortality in crude, (**B**) propensity-score-matched populations and (**C**) in-hospital mortality in crude, (**D**) propensity-score-matched populations.

The mild elevation group was further divided into two groups according to the median preoperative hs-cTn I concentration (14 ng/L). The baseline characteristics of the three groups are presented Supplementary Table S3. Each subgroup was compared to the LOD group in terms of 30-day and in-hospital mortalities. Both groups showed significantly greater 30-day mortality in the entire population (2.1% vs. 3.3%; HR 1.60; 95% CI 1.20–2.11; p = 0.001 for the below median group and 2.1% vs. 4.6%; HR 1.51; 95% CI 1.34–1.71; p < 0.001 for the above median group; Supplementary Table S4). In the propensity-score-matched population, the numerical values of the incidences showed graded elevations, but only the patients with hs-cTn concentrations above the median value were significantly different (2.6% vs. 5.0%; HR 1.93; 95% CI 1.47–2.53; p < 0.001) (Supplementary Table S4). Similar results were shown for in-hospital mortality. Both groups showed significantly greater mortality in the entire population (2.8% vs. 4.5%; HR 1.39; 95% CI 1.09–1.77; p = 0.01 for the below median group and 2.8% vs. 6.4%; HR 1.32; 95% CI 1.19–1.47; p < 0.001 for the above median group; Supplementary Table S4), but in the propensity-score-matched population, only the patients with hs-cTn concentrations above the median value showed significantly different results (3.6% vs. 6.7%; HR 1.55; 95% CI 1.23–1.97; p < 0.001) (Supplementary Table S4). Survival curves for the three groups are presented in Fig. 2.

### Threshold of preoperative hs-cTn I

**Figure 3:**
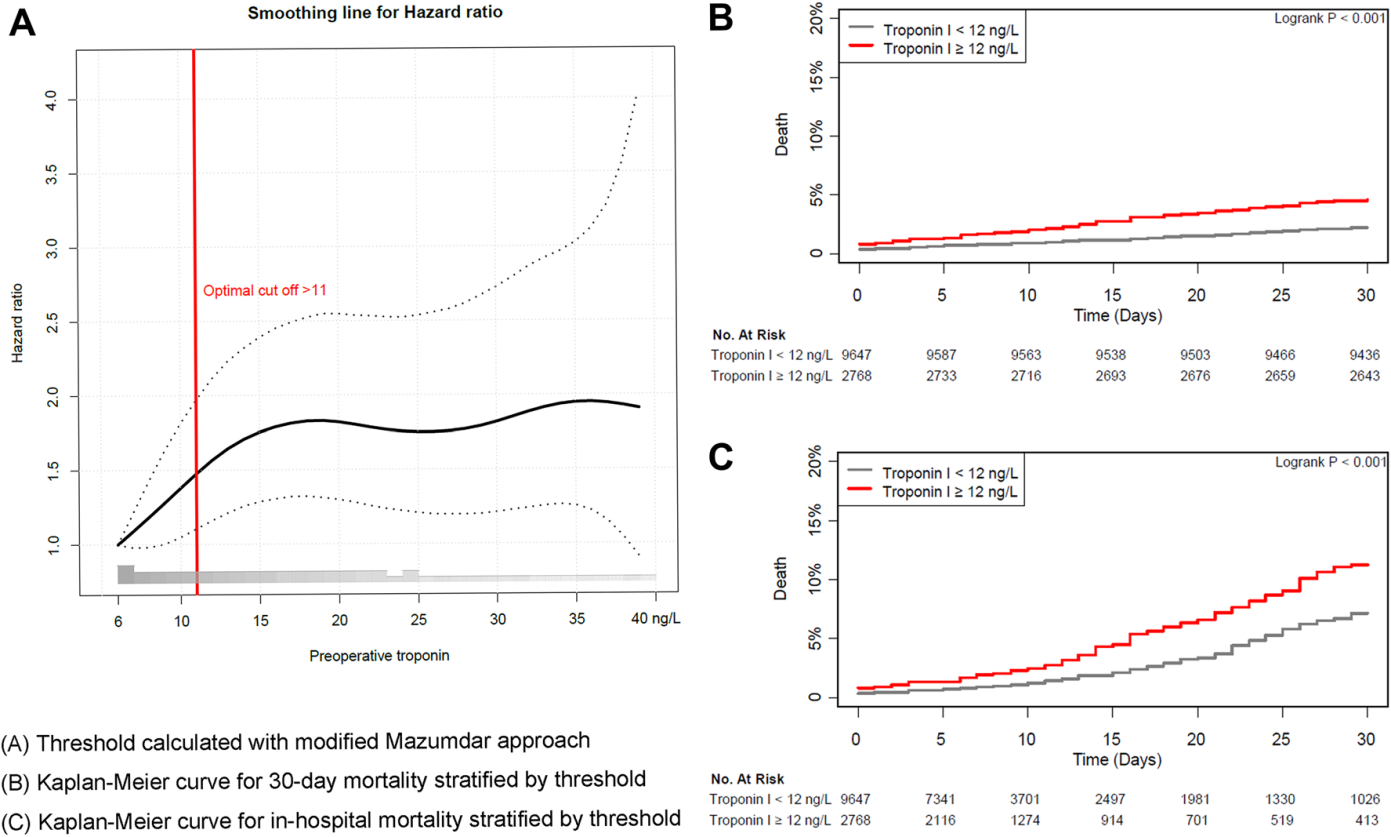
(**A**) Smoothed log-hazard ratio to calculate the threshold for preoperative high-sensitivity cardiac troponin I using a modified Mazumdar approach, (**B**) Kaplan–Meier curves for 30-day mortality according to the threshold, and (**C**) Kaplan–Meier curves for in-hospital mortality according to the threshold.

In the generation of repeated split samples, thresholds for preoperative hs-cTn I associated with 30-day mortality converged at 12 ng/L, and the estimated HR was 1.66 (IQR 1.6–1.7). The smoothed log-hazard ratio for the preoperative hs-cTn I threshold is shown in Fig. 3. By applying this threshold, 9647 (77.7%) patients were stratified into the < 12 ng/L group and 2768 (22.3%) into the ≥ 12 ng/L group (Supplementary Table S5)*.* After an adjustment, the patients in the ≥ 12 ng/L group showed a significantly increased risk of 30-day and in-hospital mortality (2.2% vs. 4.6%; HR 1.54; 95% CI 1.23–1.93; p < 0.001 and 3.0% vs. 6.3%; HR 1.28; 95% CI 1.05–1.55; p = 0.01, respectively; Table 3). Survival curves according to the calculated threshold are demonstrated in Fig. 3.

**Table 3:** Clinical outcomes according to the calculated threshold of hs-cTn I ≥ 12 ng/L.

	Univariate analysis				Multivariate analysis	
	Hs-cTn I < 12 ng/L (N = 9647)	Hs-cTn I ≥ 12 ng/L (N = 2768)	Unadjusted HR (95% CI)	p-Value	^a^Adjusted HR (95% CI)	p-Value
30-day mortality	215 (2.2)	127 (4.6)	1.88 (1.51–2.33)	< 0.001	1.54 (1.23–1.93)	< 0.001
In-hospital mortality	291 (3.0)	175 (6.3)	1.53 (1.28–1.85)	< 0.001	1.28 (1.05–1.55)	0.01
MINS	853/5007 (17.0)	631/1608 (39.2)				

Data are presented as n (%) or mean (± standard deviation).

*HR* hazard ration; *CI* confidential interval; *hs-cTn* high-sensitivity cardiac troponin; *MINS* myocardial injury after noncardiac surgery.

^a^Covariates include sex, chronic kidney disease, previous stroke, preoperative hemoglobin, statin, beta-blocker, calcium channel blocker, renin–angiotensin–aldosterone system inhibitor, emergency surgery, general anesthesia, operative duration, and intraoperative inotropic use and red cell transfusion.

### Postoperative hs-cTn I & MINS

**Figure 4:**
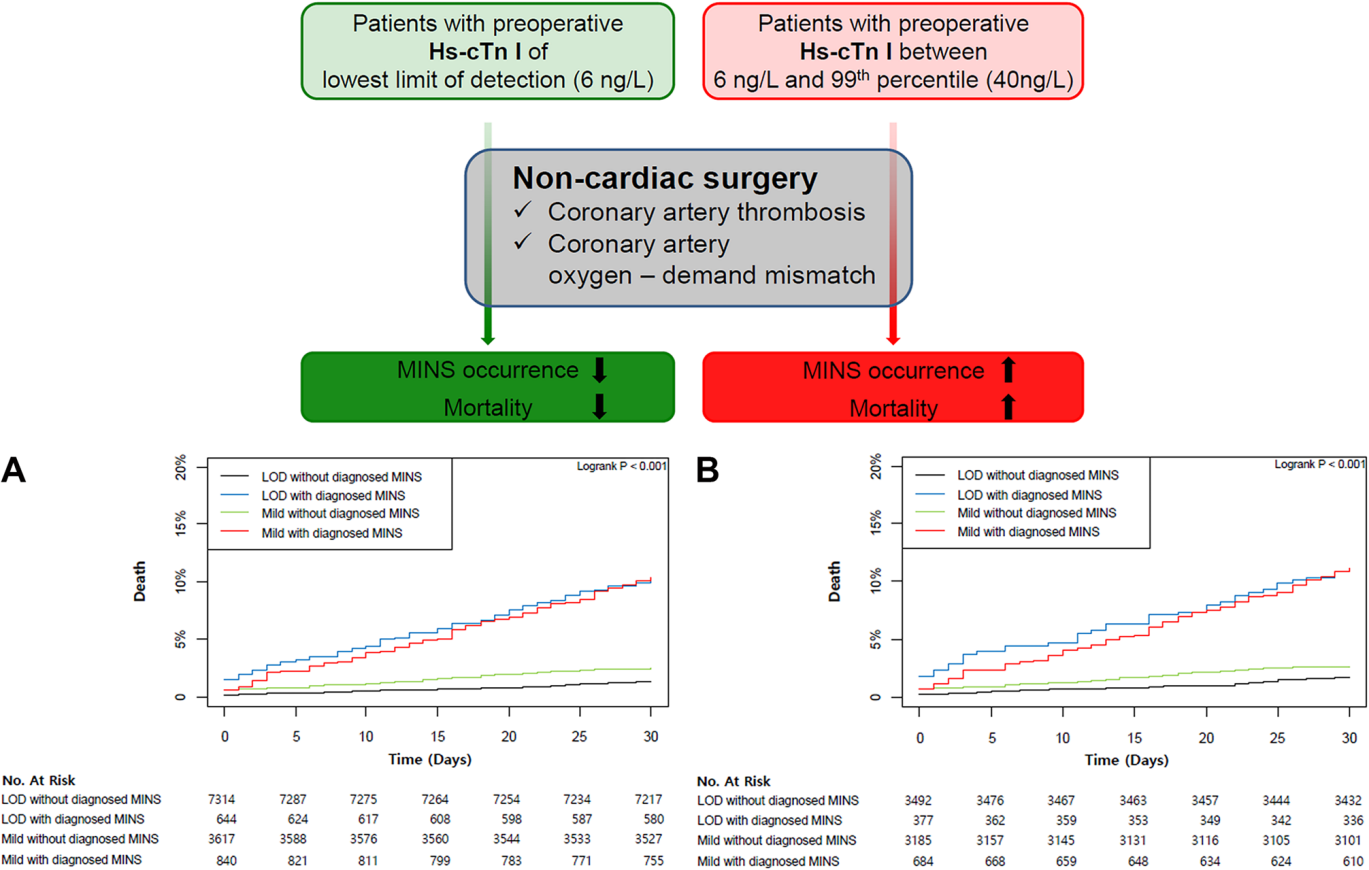
Kaplan–Meier curves for 30-day mortality according to diagnosed MINS; (**A**) crude and (**B**) propensity-score-matched population.

The peak hs-cTn I concentrations within 30 days post-surgery were available in 51.3% (4086/7958) of patients in the LOD group and 56.7% (2529/4457) in the mild elevation group. Comparisons of the baseline characteristics of the patients according to the availability of postoperative hs-cTn measurements are presented in the Supplementary Table S6*.* In patients with postoperative hs-cTn measurements, the median durations from surgery to the peak hs-cTn I concentration were 0 day (IQR 0–1) in the LOD group and 1 day (IQR 0–3) in the mild elevation group (p < 0.001). The peak concentration was significantly greater in the mild elevation group, as was the incidence of MINS in the analysis regarding the patients without postoperative hs-cTn as not having MINS. (242 ng/L vs. 1179 ng/L; p < 0.001 and 644 (8.1%) vs. 840 (18.8%); p < 0.001, Table 2). The two groups in the primary analysis were divided according to the diagnosis of MINS, and four groups were generated from the entire and propensity-score-matched populations (Supplementary Tables S7 and S8). The 30-day mortality was also increased for those with preoperative hs-cTn above the LOD in the comparison between the patients without MINS (1.4% vs. 2.5%; HR 1.87; 95% CI 1.41–2.49; p < 0.001), but the increase in 30-day mortality was more pronounced, regardless of preoperative hs-cTn concentration, for the patients who were diagnosed with MINS compared to the LOD group without MINS (1.4% vs. 10.1%; HR 7.83; 95% CI 5.72–10.70; p < 0.001 in the LOD group and 1.4% vs. 10.4%; HR 8.01; 95% CI 6.00–10.68; p < 0.001 in the mild elevation group; Table 4). Similar results were found in the propensity-score-matched population (1.7% vs. 2.7%; HR 1.61; 95% CI 1.16–2.25; p = 0.005, 1.7% vs. 11.2%; HR 7.06; 95% CI 4.76–10.47; p < 0.001, and 1.7% vs. 11.1%; HR 6.99; 95% CI 4.97–9.84; p < 0.001, respectively; Table 4). The same analyses comparing the four groups were conducted in patient with available postoperative hs-cTn I measurement (Supplementary Tables S9 and S10) and showed the similar results (1.7% vs. 2.9%; HR 1.76; 95% CI 1.10–2.81; p = 0.02, 1.7% vs. 11.2%; HR 7.11; 95% CI 4.44–11.38; p < 0.001, and 1.7% vs. 11.1%; HR 7.05; 95% CI 4.59–10.81; p < 0.001, respectively; Table 5). The survival curves of the four groups are presented in Fig. 4.

**Table 4:** Clinical outcomes according to the diagnosis of myocardial injury after noncardiac surgery in the entire population.

	Before propensity score matching				After propensity score matching			
	LOD		Mild elevation		LOD		Mild elevation	
	No diagnosed MINS (N = 7314)	Diagnosed MINS (N = 644)	No diagnosed MINS (N = 3617)	Diagnosed MINS (N = 840)	No diagnosed MINS (N = 3484)	Diagnosed MINS (N = 385)	No diagnosed MINS (N = 3185)	Diagnosed MINS (N = 684)
30-day mortality	99 (1.4)	65 (10.1)	91 (2.5)	87 (10.4)	58 (1.7)	43 (11.2)	85 (2.7)	76 (11.1)
HR (95%CI)	1 [Reference]	7.83 (5.72–10.7)	1.87 (1.41–2.49)	8.01 (6.00–10.68)	1 [Reference]	7.06 (4.76–10.47)	1.61 (1.16–2.25)	6.99 (4.97–9.84)
p-Value		< 0.001	< 0.001	< 0.001		< 0.001	0.005	< 0.001
In-hospital mortality	136 (1.9)	84 (13.0)	126 (3.5)	120 (14.3)	81 (2.3)	57 (14.8)	115 (3.6)	106 (15.5)
HR (95%CI)	1 [Reference]	3.34 (2.52–4.41)	1.49 (1.17–1.91)	4.01 (3.12–5.16)	1 [Reference]	3.30 (2.33–4.67)	1.34 (1.00–1.78)	3.82 (2.84–5.14)
p-Value		< 0.001	0.001	< 0.001		< 0.001	0.05	< 0.001

Data are presented as n (%).

30-day peak hs-cTn I was available in 4086/7958 (51.3%) in LOD and 2529/4457 (56.7%) in mild elevation groups of the entire population.

30-day peak hs-cTn I was available in 2142/3869 (55.4%) in LOD and 2211/3869 (57.1%) in mild elevation groups of the propensity score matched population.

*LOD* limit of detection; *HR* hazard ration; *CI* confidential interval; *MINS* myocardial injury after noncardiac surgery.

**Table 5:** Clinical outcomes according to the diagnosis of myocardial injury after noncardiac surgery in patients with postoperative hs-cTn I.

	Before propensity score matching				After propensity score matching			
	LOD		Mild elevation		LOD		Mild elevation	
	No diagnosed MINS (N = 3442)	Diagnosed MINS (N = 644)	No diagnosed MINS (N = 1689)	Diagnosed MINS (N = 840)	No diagnosed MINS (N = 1757)	Diagnosed MINS (N = 385)	No diagnosed MINS (N = 1527)	Diagnosed MINS (N = 684)
30-day mortality	52 (154)	65 (10.1)	47 (2.8)	87 (10.4)	29 (1.7)	43 (11.2)	44 (2.9)	76 (11.1)
HR (95%CI)	1 [Reference]	6.99 (4.86–10.07)	1.86 (1.25–2.76)	7.16 (5.08–10.10)	1 [Reference]	7.11 (4.44–11.38)	1.76 (1.10–2.81)	7.05 (4.59–10.81)
p-Value		< 0.001	0.002	< 0.001		< 0.001	0.02	< 0.001
In-hospital mortality	77 (2.2)	84 (13.0)	66 (3.9)	120 (14.3)	46 (2.6)	57 (14.8)	61 (4.0)	106 (15.5)
HR (95%CI)	1 [Reference]	2.59 (1.88–3.56)	1.36 (0.98–1.90)	3.12 (2.32–4.17)	1 [Reference]	2.61 (1.75–3.90)	1.25 (0.85–1.84)	3.03 (2.13–4.31)
p-Value		< 0.001	0.07	< 0.001		< 0.001	0.25	< 0.001

Data are presented as n (%).

*LOD* limit of detection; *HR* hazard ration; *CI* confidential interval; *hs-cTn* high-sensitivity cardiac troponin; *MINS* myocardial injury after noncardiac surgery.

## Discussion

The main findings of this study are as follows: (1) preoperative hs-cTn concentrations above the lowest LOD but below the 99th-percentile URL were significantly associated with early mortality in patients, (2) this association was consistent regardless of the occurrence of MINS, (3) the incidence of MINS was also increased in patients with preoperative hs-cTn above the lowest LOD, and the patients who were diagnosed with MINS had an enormously increased risk of postoperative mortality regardless of preoperative hs-cTn, and (4) the calculated threshold at which mortality increased was a preoperative hs-cTn concentration of 12 ng/L, well below the 99th-percentile URL. These findings indicate that the risk of mortality may differ according to the preoperative hs-cTn concentration even when it is below the 99th-percentile URL. Based on these findings, we suggest that preoperative hs-cTn concentration below the 99th-percentile URL could be used to predict noncardiac surgical risk that might otherwise be overlooked.

In clinical practice, risk assessment of a patient’s cardiac troponin concentration has been generally comprised a binary “rule in” or “rule out” threshold at the 99th-percentile URL. This cut-off has allowed an early confident “ruling out” of myocardial infarction^18^, but “ruling in” of myocardial infarction solely on the basis elevated cardiac troponin often leads to a misinterpretation^19^. On the other hand, postoperative troponin elevation has shown a robust association with mortality, regardless of ischemic symptoms^1–3,9^. Based on these findings, the diagnostic criteria for MINS were established using postoperative cardiac troponin concentrations above the 99th-percentile URL^4^. However, for the preoperative period, cardiac troponin concentrations below this limit have generally been defined as normal without accurately evaluating the associated risk. According to our results, uniformly applying this threshold for perioperative cardiac troponin may also lead to a misinterpretation. These findings are in line with studies of other biomarkers showing associations between the baseline measurements within the normal range and adverse outcomes in stable patients^12–14^.

Another issue to be discussed is the generalizability of the 99th-percentile URL and whether a preoperative cardiac troponin threshold other than the 99th-percentile URL is needed to “rule in” or “rule out” patients at risk. As mentioned above, the 99th-percentile URL was previously well validated to rule out myocardial infarction^18^, and was also adopted for perioperative evaluations. Based on clinical relevance shown in previous studies^3–5,20^, the Fourth Universal Definition of Myocardial Infarction also recommended using the 99th-percentile URL of any assay to define myocardial injury, but at the same time it specifically highlighted myocardial injury as discrete from myocardial infarction^5^. Moreover, some investigators recently argued that a lower cut-off should be applied even for ruling out acute myocardial infarction^21^. In addition, the 99th-percentile URLs provided by immunoassay manufacturers are derived from a limited number of apparently healthy individuals^22^, and several factors such as age, sex, kidney and heart function, and the presence of inflammation that are known to affect troponin level are not considered^17,23^. Therefore, clinical application of the same limit to surgical patients requires closer scrutiny, because surgical patients are likely to show discrete characteristics from the reference population.

A mechanism for the increased mortality in the mild elevation group can be inferred from our additional analysis involving the occurrence of MINS. The incidence of MINS, as well as peak postoperative troponin concentration, was greater in the mild elevation group, and the increased mortality of the mild elevation group was mostly driven by the patients who were diagnosed with MINS. Furthermore, the increased risk of mortality was more notable in patients diagnosed with MINS regardless of preoperative cardiac troponin level. These findings provide a clue that the development of MINS still plays a key role for mortatlity, and the patients with cardiac troponin above the LOD were more likely to develop MINS. However, the mild elevation group consistently showed increased mortality in patients without MINS, and in patients with MINS, the prognosis did not seem to differ according to mild elevation of preoperative hs-cTn. These findings have clinical implications for extending the use of hs-cTn in perioperative risk management. The current guidelines aimed at preventing or detecting MINS, and the treatments are also focused on postoperative cardiac troponin concentrations^24,25^, because secondary prevention with cardiovascular drugs was shown to be beneficial and cost-effective^4,6,7,26^. However, previous evidences also indicated incremental increase of mortality according to preoperative cardiac troponin elevation^8^, and supported the assessment of both pre- and postoperative in cardiac troponin^9,10^. As an extension of these studies, our results indicate preventive measures should be considered for patients with preoperative cardiac troponin below the 99th-percentile URL.

In further analyses, the mild elevation group was divided into two groups according to the median preoperative hs-cTn concentration. Because only the patients with troponin concentrations above the median value consistently had increased mortality after propensity score matching, we estimated the threshold preoperative hs-cTn I concentration where the risk for mortality significantly increased. Although mortality significantly differed according to the estimated threshold in this study, the clinical relevance of this threshold needs further evaluation after inclusion of patients with hs-cTn over the 99th-percentile URL.

Our results have the following limitations. First, as a single-center retrospective study, our results may have been affected by confounding factors. Despite rigorous adjustments in the multivariate and propensity-score-matched analyses and sensitivity analysis, it is possible the result were impacted by unmeasured variables related to postoperative morbidities or oxygen/supply demand mismatch. Second, perioperative hs-cTn measurement was not included as a routine practice at our institution, and it could also have been measured at the discretion of attending clinician despite the institutional indication. And also, cardiac troponin was generally measured in patients with risk or ischemic symptom, so the median duration from the surgery to the peak cardiac troponin level was longer in the mild elevation group. Consequently, the results of the current study may have been biased. Moreover, postoperative serum troponin measurements were available for only about half of the patients, and the patients without postoperative measurements were considered not to have MINS. Although those patients were likely to be asymptomatic with lower cardiac risk, the results might have been biased, and further studies with a detailed protocol for cardiac troponin measurement are needed. Despite these limitations, this is the first study on the association between hs-cTn above the lowest LOD but below the 99th-percentile URL and 30-day mortality using a robust propensity score matching. Our results provide evidence that clinicians should be cautious when interpreting serum hs-cTn concentrations below the 99th-percentile URL as “normal” in the preoperative risk assessment for noncardiac surgery patients.

## Methods

### Study population and data curation

This study was approved by the Institutional Review Board of the Samsung Medical Center, Seoul, Korea (SMC 2019-08-048) on 12th July 2019 and was conducted according to the principles of the Declaration of Helsinki. The requirement for individual informed consent was waived by the Institutional Review Board of the Samsung Medical Center considering the minimal risk to the patients. From January 2010 to April 2019, all consecutive adult patients who underwent noncardiac surgery with serum hs-cTn I concentrations below the 99th-percentile URL during their preoperative evaluation at our institution were enrolled. All data in this study were curated using “Clinical Data Warehouse Darwin-C.” It is an electronic system built for investigators to search and retrieve de-identified medical records from the institutional archive system containing clinical data from more than 4 million patients, including data from more than 2 million surgeries, 900 million laboratory findings, and 200 million prescriptions. In this system, mortalities outside of our institution were consistently updated and validated against the National Population Registry of the Korea National Statistical Office using a unique personal identification number when available. The patients were divided into two groups according to preoperative hs-cTn I concentration: (1) the LOD group (having [hs-cTn I] < 6 ng/L) and (2) the mild elevation group (having [hs-cTn I] < 40 ng/L).

### Study outcomes and definitions

The primary outcome was 30-day mortality, and in-hospital mortality was also compared between the two study groups. The peak hs-cTn I concentration within 30 days after surgery was compared in patients with available postoperative hs-cTn I measurements. Since preoperative hs-cTn level of our study population was below the 99th-percentile URL, postoperative hs-cTn I elevation above the URL within 30 days was all regarded as MINS, and patients without elevated postoperative hs-cTn I were regarded as not having been diagnosed with MINS. The baseline characteristics were derived from the preoperative evaluation sheets of the patients’ electronic medical records. Surgical risk was stratified according to the 2014 European Society of Cardiology/Anesthesiology (ESC/ESA) guidelines^24^.

### Preoperative evaluation and Hs-cTn I measurement

Preoperative evaluation at our institution starts at the outpatient department and includes routine blood tests, plain chest film and echocardiography within the 30 days prior to the surgery. If necessary, further evaluations are made in a multidisciplinary manner at the anesthesiologist’s discretion.

Perioperative hs-cTn I was recommended for the patients with moderate- to severe cardiovascular risk or for those undergoing intermediate- to high-risk surgery according to the guidelines^24^, but it was also measured at the discretion or request of an attending clinician in patients with mild risk. An automated analyzer (Advia Centaur XP, Siemens Healthcare Diagnostics, Erlangen, Germany) was used throughout the entire study period to assess the results of the highly sensitive immunoassay. The lowest LOD was 6 ng/L, and the 99th-percentile URL of 40 ng/L was provided by the manufacturer.

### Statistical analyses

Baseline characteristics were presented as numbers and percentages for categorical variables and as the mean ± standard deviation or median with IQR for continuous variables. We used parametric or non-parametric tests as appropriate to compare differences in baseline characteristics in the preliminary analyses. Kaplan–Meier survival curves were constructed and compared with the log-rank test. Variables for the multivariate Cox regression model were selected based on a standardized mean difference larger than 10%. To further reduce selection bias while maintaining a balance between the confounding variables of the two groups, we used propensity score matching on preoperative variables. An appropriate balance between the groups with an absolute SMD of less than 10% for all pre-and intraoperative variables suggested successful propensity matching. In the propensity-score-matched population, we computed HR and 95% CI for outcomes using a multivariate Cox regression model with adjustments for intraoperative variables. The sensitivity analysis for the primary outcome was conducted by estimating the potential impact of unmeasured confounders, and the power of the study regarding the sample size was calculated using Spearman's rank correlation^27,28^.

We also calculated a threshold of preoperative hs-cTn I concentration associated with 30-day mortality using a modified Mazumdar approach^29^. We selected covariates for multiple logistic regression using a stepwise Akaike information criterion method, and chose an initial threshold to maximize the log-likelihood of the estimated multiple model. For the sensitivity analysis of the calculated threshold, split sample generation was repeated 500 times to obtain the distribution of thresholds and HRs. Based on the results of the Cox proportional hazards model, we also determined the attributable fraction for each variable that was independently associated with 30-day mortality^30^. This is a measure that represents the proportional reduction in mortality within a population that would occur if the variable was causal and the incidence of the variable was reduced to none. All statistical analyses were performed with R 4.0.0 (Vienna, Austria; https://www.R-project.org/). All tests were two-tailed, and a p < 0.05 was considered statistically significant.

## Conclusions

In patients undergoing noncardiac surgery, preoperative hs-cTn I concentrations above the lowest LOD but below the 99th-percentile URL were significantly associated with 30-day mortality. A larger registry-based or cohort study is needed to confirm this finding.

## Supplementary information

Supplementary file1

## Abbreviations

CI: Confidence interval
hs-cTn: High-sensitivity cardiac troponin
HR: Hazard ratio
IQR: Interquartile range
URL: Upper reference limit
