# Supplementary material for: Preoperative cardiac troponin below the 99th-percentile upper reference limit and 30-day mortality after noncardiac surgery
Source: Sci Rep. 2020 Oct 12;10:17007. doi: 10.1038/s41598-020-72853-3 (PMC7550329; doi:10.1038/s41598-020-72853-3)
Supplement: Supplementary file 1 — Supplementary file1 [file 41598_2020_72853_MOESM1_ESM.docx]

**Preoperative Cardiac Troponin Below the 99^th^-Percentile upper Reference Limit and 30-Day Mortality after Noncardiac Surgery**

**Short title: Normal Preoperative Troponin in Noncardiac Surgery**

Jungchan Park, MD,^a^ Cheol Won Hyeon, MD,^b^ Seung-Hwa Lee, MD,^b^ Sangmin Maria Lee, MD, PhD,^a^ Junghyun Yeo, MD,^a^ Kwangmo Yang, MD,^c^ Jeong Jin Min, MD, PhD,^a^ Jong Hwan Lee, MD, PhD,^a^ Jeong Hoon Yang, MD, PhD,^b^ Young Bin Song, MD, PhD,^b^ Joo-Yong Hahn, MD, PhD,^b^ Seung-Hyuk Choi, MD, PhD,^b^ Jin-Ho Choi, MD, PhD,^b,d^ and Hyeon-Cheol Gwon, MD, PhD^b^

^a^ Department of Anesthesiology and Pain Medicine, Samsung Medical Center, Sungkyunkwan University School of Medicine, Seoul, Korea

^b^ Division of Cardiology, Department of Medicine, Heart Vascular Stroke Institute, Samsung Medical Center, Sungkyunkwan University School of Medicine, Seoul, Korea

^c^ Centers for Health Promotion, Samsung Medical Center, Sungkyunkwan University School of Medicine, Seoul, Korea

^d^ Department of Emergency Medicine, Samsung Medical Center, Sungkyunkwan University School of Medicine, Seoul, Korea

The first two authors contributed equally to this work (J.P, C.W.H).

**Corresponding author:** Seung-Hwa Lee, MD, Division of Cardiology, Department of Medicine, Heart Vascular Stroke Institute, Samsung Medical Center, Sungkyunkwan University School of Medicine, 81 Irwon-ro, Gangnam-gu, Seoul, Korea.

Tel: +82-2-3410-3214; Fax: +82-2-3410-2849

E-mail address: shuaaa.lee@samsung.com

**Supplemental table 1. Sensitivity analysis of the effect of an unmeasured confounder on hazard ratio of minor elevation for mortality compared with the lowest limit of detection**

|  |  | OR*_ZY_*_\|_*_X_* | | | | | |
| --- | --- | --- | --- | --- | --- | --- | --- |
|  |  | 1.5 | 2 | 2.5 | 3 | 3.5 | 4 |
| OR_zx_ | 0.3 | 1.90 (1.47-2.46) | 2.07 (1.60-2.68) | 2.21 (1.71-2.86) | 2.32 (1.79-3.00) | 2.45 (1.90-3.17) | 2.51 (1.94-3.25) |
|  | 0.4 | 1.81 (1.40-2.33) | 1.92 (1.49-2.48) | 2.02 (1.57-2.61) | 2.11 (1.63-2.72) | 2.16 (1.68-2.79) | 2.23 (1.73-2.87) |
|  | 0.5 | 1.74 (1.36-2.25) | 1.83 (1.42-2.36) | 1.90 (1.48-2.45) | 1.96 (1.52-2.52) | 2.02 (1.57-2.60) | 2.04 (1.59-2.63) |
|  | 0.6 | 1.70 (1.32-2.18) | 1.76 (1.37-2.26) | 1.81 (1.41-2.32) | 1.86 (1.44-2.38) | 1.88 (1.46-2.41) | 1.92 (1.49-2.46) |
|  | 0.7 | 1.67 (1.30-2.14) | 1.71 (1.33-2.19) | 1.75 (1.36-2.24) | 1.77 (1.38-2.28) | 1.79 (1.40-2.30) | 1.82 (1.42-2.33) |

Prevalence of unmeasured confounder = 40%

Numbers represent HRs (including 95% CIs).

OR, odds ratio; HR, hazard ratio; X: dichotomous exposure measure, y dichotomous outcome measure, z : potential dichotomous confounder.

OR_ZX_ indicates the association (OR) between the unmeasured confounder and minor elevation.

OR_ZY|X_ indicates the association (OR) between the unmeasured confounder and mortality conditional on exposure status.

**Supplemental Table 2. Predictors of 30-day mortality among variables in multivariable model**

|  | 30-day mortality/total patients | 30-day mortality (95% CI) | Adjusted HR (95% CI) | AF* |
| --- | --- | --- | --- | --- |
| Emergency | 142/9515 | 1.5(1.2-1.8) | Reference | 40.9 |
| No emergency | 200/2900 | 6.9(6.0-7.9) | 3.48 (2.78-4.36) |  |
| Minor elevation | 164/7958 | 2.1 (1.8-2.4) | Reference | 21.2 |
| No minor elevation | 178/4457 | 4.0 (3.4-4.6) | 1.73 (1.39-2.16) |  |
| ESC/ESA surgical risk |  |  | Reference |  |
| *High* | 38/1433 | 2.7(1.9-3.6) |  | 10.6 |
| *Intermediate* | 195/7387 | 2.6(2.3-3.0) | 1.71 (1.08-2.69) | 7.3 |
| *Mild* | 109/3595 | 3.0(2.5-3.6) | 0.89 (0.70-1.14) |  |
| General anesthesia | 116/2783 | 4.2 (3.5-5.0) | Reference | 10 |
| No general anesthesia | 226/9632 | 2.3(2.1-2.7) | 0.87 (0.68-1.11) |  |
| RAAS inhibitor | 262/8178 | 3.2(2.8-3.6) | Reference | 9.9 |
| No RAAS inhibitor | 80/4237 | 1.9(1.5-2.3) | 0.69 (0.51-0.94) |  |
| Chronic kidney disease | 334/11517 | 2.9(2.6-3.2) | Reference | 7.9 |
| No chronic kidney disease | 8/898 | 0.9 (0.4-1.7) | 0.22 (0.11-0.45) |  |
| Beta-blocker | 275/9016 | 3.1(2.7-3.4) | Reference | 7.1 |
| No beta-blocker | 67/3399 | 2.0(1.5-2.5) | 0.73 (0.53-0.99) |  |
| Calcium channel blocker | 214/7649 | 2.8(2.4-3.2) | Reference | 6.9 |
| No calcium channel blocker | 128/4766 | 2.7(2.2-3.2) | 1.24 (0.92-1.66) |  |
| Diabetes | 262/8963 | 2.9 (2.6-3.3) | Reference | 3.5 |
| No diabetes | 80/3452 | 2.3(1.8-2.9) | 0.86 (0.66-1.13) |  |
| Previous stroke | 329/11533 | 2.9(2.6-3.2) | Reference | 3.1 |
| No previous stroke | 13/882 | 1.5(0.8-2.4) | 0.54 (0.31-0.95) |  |
| Antiplatelet agent | 262/8262 | 3.2(2.8-3.6) | Reference | 2.8 |
| No antiplatelet agent | 80/4153 | 1.9 (1.5-2.4) | 0.89 (0.66-1.20) |  |
| Coronary artery disease | 316/10802 | 2.9 (2.6-3.3) | Reference | 1.1 |
| No coronary artery disease | 26/1613 | 1.6 (1.1-2.3) | 0.87 (0.56-1.34) |  |
| Hypertension | 143/4676 | 3.1(2.6-3.6) | Reference | 0.9 |
| No hypertension | 199/7739 | 2.6(2.2-2.9) | 1.01 (0.73-1.40) |  |
| Arrhythmia | 322/11624 | 2.8(2.5-3.1) | Reference | 0.4 |
| No arrhythmia | 20/791 | 2.5 (1.6-3.8) | 1.08 (0.68-1.71) |  |

AF indicates attributable fraction; HR, hazard ration; CI, confidential interval; ESC, European Society of Cardiology; ESA, European Society of Anaesthesiology; RAAS, renin-angiotensin-aldosterone system.

The AF is a measure that represents the proportional reduction in mortality within a population that would occur if the variable was absent, provided that a causal relation existed between that variable and 30-day mortality. We used incidence of variable and the association between the variable and mortality to calculate the AF.

**Supplemental Table 3. Baseline characteristics 3 group stratification according to the median level of hs-cTn**

|  | Entire population | | | | Propensity-score matched population | | | |
| --- | --- | --- | --- | --- | --- | --- | --- | --- |
|  | LOD  (N=7958) | Mild elevation | | *P*-value | LOD (N=3869) | Mild elevation | | *P*-value |
|  |  | Below median (N = 2112) | Above median (N = 2345) |  |  | Below median (N = 1774) | Above median (N = 2095) |  |
| Preoperative hs-cTn I, ng/L | 6 | 10 (±2) | 23 (±7) |  | 6 | 9 (±2) | 22 (±7) |  |
| ***Preoperative variables*** |  |  |  |  |  |  |  |  |
| Male | 4432 (55.7) | 1219 (57.7) | 1384 (59.0) | 0.01 | 2217 (57.3) | 1020 (57.5) | 1213 (57.9) | 0.91 |
| Age | 58.0 (±18.0) | 63.6 (±16.5) | 64.2 (±17.4) | <0.001 | 62.6 (±16.0) | 63.0 (±16.6) | 63.0 (±18.0) | 0.61 |
| Hypertension | 4417 (55.5) | 1467 (69.5) | 1855 (79.1) | <0.001 | 2722 (70.4) | 1178 (66.4) | 1563 (74.6) | <0.001 |
| Diabetes | 1924 (24.2) | 665 (31.5) | 863 (36.8) | <0.001 | 1196 (30.9) | 511 (28.8) | 679 (32.4) | 0.05 |
| Coronary artery disease | 802 (10.1) | 335 (15.9) | 476 (20.3) | <0.001 | 585 (15.1) | 237 (13.4) | 341 (16.3) | 0.04 |
| Chronic kidney disease | 268 (3.4) | 184 (8.7) | 446 (19.0) | <0.001 | 264 (6.8) | 78 (4.4) | 186 (8.9) | <0.001 |
| Previous stroke | 485 (6.1) | 157 (7.4) | 240 (10.2) | <0.001 | 321 (8.3) | 125 (7.0) | 188 (9.0) | 0.09 |
| Arrhythmia | 339 (4.3) | 165 (7.8) | 287 (12.2) | <0.001 | 287 (7.4) | 119 (6.7) | 184 (8.8) | 0.04 |
| Current smoking | 663 (8.3) | 158 (7.5) | 152 (6.5) | 0.01 | 271 (7.0) | 142 (8.0) | 142 (6.8) | 0.29 |
| Preoperative hemoglobin, g/dl | 12.5 (±2.0) | 12.1 (±2.1) | 11.4 (±2.1) | <0.001 | 11.9 (±2.1) | 12.2 (±2.0) | 11.7 (±2.1) | <0.001 |
| Medication |  |  |  |  |  |  |  |  |
| Antiplatelet agent | 2232 (28.0) | 835 (39.5) | 1086 (46.3) | <0.001 | 1453 (37.6) | 628 (35.4) | 846 (40.4) | 0.01 |
| Statin | 2165 (27.2) | 722 (34.2) | 915 (39) | <0.001 | 1263 (32.6) | 554 (31.2) | 723 (34.5) | 0.09 |
| Beta-blocker | 1689 (21.2) | 685 (32.4) | 1025 (43.7) | <0.001 | 1262 (32.6) | 494 (27.8) | 772 (36.8) | <0.001 |
| Calcium channel blocker | 2586 (32.5) | 910 (43.1) | 1270 (54.2) | <0.001 | 1725 (44.6) | 697 (39.3) | 1006 (48.0) | <0.001 |
| RAAS inhibitor | 2236 (28.1) | 837 (39.6) | 1164 (49.6) | <0.001 | 1520 (39.3) | 633 (35.7) | 887 (42.3) | <0.001 |
| ESC/ESA surgical risk |  |  |  | <0.001 |  |  |  | 0.02 |
| *Mild* | 2441 (30.7) | 576 (27.3) | 578 (24.6) |  | 1112 (28.7) | 491 (27.7) | 551 (26.3) |  |
| *Intermediate* | 4554 (57.2) | 1298 (61.5) | 1535 (65.5) |  | 2265 (58.5) | 1078 (60.8) | 1319 (63.0) |  |
| *High* | 963 (12.1) | 238 (11.3) | 232 (9.9) |  | 492 (12.7) | 205 (11.6) | 225 (10.7) |  |
| Operation type |  |  |  | <0.001 |  |  |  | <0.001 |
| *Vascular* | 982 (12.3) | 290 (13.7) | 382 (16.3) |  | 489 (12.6) | 227 (12.8) | 280 (13.4) |  |
| *Orthopedics* | 2371 (29.8) | 604 (28.6) | 774 (33.0) |  | 1198(31.0) | 503 (28.4) | 689 (32.9) |  |
| *Abdominal* | 984 (12.4) | 334 (15.8) | 372 (15.9) |  | 590 (15.2) | 272 (15.3) | 324 (15.5) |  |
| *Thoracic* | 22 (0.3) | 5 (0.2) | 3 (0.1) |  | 7 (0.2) | 5 (0.3) | 3 (0.1) |  |
| *Neuro* | 2437 (30.6) | 532 (25.2) | 379 (16.2) |  | 1007 (26.0) | 486 (27.4) | 390 (18.6) |  |
| *Otolaryngology, Eye* | 707 (8.9) | 212 (10.0) | 274 (11.7) |  | 341 (8.8) | 174 (9.8) | 255 (12.2) |  |
| *Urology, gynecology* | 455 (5.7) | 135 (6.4) | 161 (6.9) |  | 237 (6.1) | 107 (6.0) | 154 (7.4) |  |
| Emergency | 1706 (21.4) | 519 (24.6) | 675 (28.8) | <0.001 | 1004 (25.9) | 412 (23.2) | 600 (28.6) | 0.001 |
| General anesthesia | 6449 (81.0) | 1577 (74.7) | 1606 (68.5) | <0.001 | 2902 (75.0) | 1373 (77.4) | 1532 (73.1) | 0.01 |
| ****Intraoperative variables*** |  |  |  |  |  |  |  |  |
| Operation duration, minute | 161.0 (±142.1) | 146.9 (±133.5) | 133.6 (±125.2) | <0.001 | 157.0 (±143.6) | 150.6 (±134.4) | 140.7 (±131.0) | <0.001 |
| Inotropic drug requirement | 2798 (35.2) | 785 (37.2) | 966 (41.2) | <0.001 | 1487 (38.4) | 660 (37.2) | 851 (40.6) | 0.05 |
| Red blood cell transfusion | 2978 (37.4) | 782 (37.0) | 724 (30.9) | <0.001 | 1374 (35.5) | 678 (38.2) | 691 (33.0) | 0.07 |

Data are presented as n (%) or mean (±standard deviation)

LOD indicates limit of detection; hs-cTn, high-sensitivity cardiac troponin; RAAS, renin-angiotensin-aldosterone system; ESC, European Society of Cardiology; ESA, European Society of Anaesthesiology.

**Supplemental Table 4. Clinical outcomes of 3 group stratifications according to the median level of hs-cTn**

|  | Entire population | | | Propensity-score matched population | | |
| --- | --- | --- | --- | --- | --- | --- |
|  | LOD (N=7958) | Mild elevation | | LOD (N=3869) | Mild elevation | |
|  |  | Below median  (N=2112) | Above median  (N=2345) |  | Below median  (N=1774) | Above median  (N=2095) |
| 30-day mortality | 164 (2.1) | 69 (3.3) | 109 (4.6) | 101 (2.6) | 57 (3.2) | 104 (5.0) |
| HR (95%CI) | 1 [Reference] | 1.60 (1.20-2.11) | 1.51 (1.34-1.71) | 1 [Reference] | 1.23 (0.89-1.71) | 1.93 (1.47-2.53) |
| P-value |  | 0.001 | <0.001 |  | 0.20 | <0.001 |
| In-hospital mortality | 220 (2.8) | 96 (4.5) | 150 (6.4) | 138 (3.6) | 80 (4.5) | 141 (6.7) |
| HR (95%CI) | 1 [Reference] | 1.39 (1.09-1.77) | 1.32 (1.19-1.47) | 1 [Reference] | 1.16 (0.88-1.52) | 1.55 (1.23-1.97) |
| *P*-value |  | 0.01 | <0.001 |  | 0.30 | <0.001 |
|  |  |  |  |  |  |  |
| 30-day peak hs-cTn I level, ng/L | 242 (±2862) | 1475 (±20148) | 926 (±6688) | 160 (±2579) | 914 (±16302) | 497 (±4655) |

LOD indicates limit of detection; HR, hazard ration; CI, confidential interval; hs-cTn, high-sensitivity cardiac troponin.

**Supplemental Table 5. Baseline characteristics according to the calculated threshold of hs-cTn I ≥12 ng/L**

|  | Hs-cTn I < 12ng/L (N=9647) | Hs-cTn I ≥12 ng/L (N=2768) | *P*-value |
| --- | --- | --- | --- |
| ***Preoperative variables*** |  |  |  |
| Male | 5400 (56.0) | 1635 (59.1) | 0.004 |
| Age | 58.9 (±17.8) | 64.4 (±17.2) | <0.001 |
| Hypertension | 5567 (57.7) | 2172 (78.5) | <0.001 |
| Diabetes | 2425 (251) | 1027 (37.1) | <0.001 |
| Coronary artery disease | 1051 (10.9) | 562 (20.3) | <0.001 |
| Chronic kidney disease | 388 (4.0) | 510 (18.4) | <0.001 |
| Previous stroke | 609 (6.3) | 273 (9.9) | <0.001 |
| Arrhythmia | 464 (4.8) | 327 (11.8) | <0.001 |
| Current smoking | 792 (8.2) | 181 (6.5) | 0.004 |
| Preoperative hemoglobin, g/dl | 12.4 (±2.1) | 11.4 (±2.1) | <0.001 |
| Medication |  |  |  |
| Antiplatelet agent | 2870 (29.8) | 1283 (46.4) | <0.001 |
| Statin | 2718 (28.2) | 1084 (39.2) | <0.001 |
| Beta-blocker | 2211 (22.9) | 1188 (42.9) | <0.001 |
| Calcium channel blocker | 3283 (34.0) | 1483 (53.6) | <0.001 |
| RAAS inhibitor | 2888 (29.9) | 1349 (48.7) | <0.001 |
| ESC/ESA surgical risk |  |  | <0.001 |
| *Mild* | 2908 (30.1) | 687 (24.8) |  |
| *Intermediate* | 5591 (58.0) | 1796 (64.9) |  |
| *High* | 1148 (11.9) | 285 (10.3) |  |
| Operation type |  |  | <0.001 |
| *Vascular* | 1203 (12.5) | 451 (16.3) |  |
| *Orthopedics* | 2845 (29.5) | 904 (32.7) |  |
| *Abdominal* | 1242 (12.9) | 448 (16.2) |  |
| *Thoracic* | 27 (0.3) | 3 (0.1) |  |
| *Neuro* | 2893 (30.0) | 455 (16.4) |  |
| *Otolaryngology, Eye* | 876 (9.1) | 317 (11.5) |  |
| *Urology, gynecology* | 561 (5.8) | 190 (6.9) |  |
| Emergency | 2121 (22.0) | 779 (28.1) | <0.001 |
| General anesthesia | 7724 (80.1) | 1908 (68.9) | <0.001 |
| ****Intraoperative variables*** |  |  |  |
| Operation duration, minute | 158.7 (±140.8) | 134.9 (±126.2) | <0.001 |
| Inotropic drug requirement | 3429 (35.5) | 1120 (40.9) | <0.001 |
| Red blood cell transfusion | 3609 (37.4) | 875 (31.6) | <0.001 |

Data are presented as n (%) or mean (±standard deviation).

hs-cTn indicates high-sensitivity cardiac troponin; RAAS, renin-angiotensin-aldosterone system; ESC, European Society of Cardiology; ESA, European Society of Anaesthesiology.

**Supplemental table 6. Baseline characteristics and mortality comparison between the patients with and without postoperative hs-cTn I**

|  | Without postoperative hs-cTn I (N=5800) | With postoperative hs-cTn I (N=6615) | *P*-value |
| --- | --- | --- | --- |
| Preoperative hs-cTn I, ng/L | 10 (±10) | 10 (±10) | < 0.001 |
| Preoperative minor elevation | 1928 (33.2) | 2529 (38.2) | < 0.001 |
| ***Preoperative variables*** |  |  |  |
| Male | 3019 (52.1) | 4016 (60.7) | <0.001 |
| Age | 56.1 (±20.1) | 63.6 (±14.7) | <0.001 |
| Hypertension | 3144 (54.2) | 4595 (69.5) | <0.001 |
| Diabetes | 1376 (23.7) | 2076 (31.4) | <0.001 |
| Coronary artery disease | 466 (8.0) | 1147 (17.3) | <0.001 |
| Chronic kidney disease | 418 (7.2) | 480 (7.3) | 0.94 |
| Previous stroke | 323 (5.6) | 559 (8.5) | <0.001 |
| Arrhythmia | 267 (4.6) | 524 (7.9) | <0.001 |
| Current smoking | 390 (6.7) | 583 (8.8) | <0.001 |
| Preoperative hemoglobin, g/dl | 12.2 (±2.1) | 12.2 (±2.1) | 0.61 |
| Medication |  |  |  |
| Antiplatelet agent | 1512 (26.1) | 2641 (39.9) | <0.001 |
| Statin | 1423 (24.5) | 2379 (36.0) | <0.001 |
| Beta-blocker | 1285 (22.2) | 2114 (32.0) | <0.001 |
| Calcium channel blocker | 2042 (35.2) | 2724 (41.2) | <0.001 |
| RAAS inhibitor | 1671 (28.8) | 2566 (38.8) | <0.001 |
| ESC/ESA surgical risk |  |  | <0.001 |
| *Mild* | 2269 (39.1) | 1326 (20.0) |  |
| *Intermediate* | 3353 (57.8) | 4034 (61.0) |  |
| *High* | 178 (3.1) | 1255 (19.0) |  |
| Operation type |  |  | <0.001 |
| *Vascular* | 447 (7.7) | 1207 (18.2) |  |
| *Orthopedics* | 1541 (26.6) | 2208 (33.4) |  |
| *Abdominal* | 891 (15.4) | 799 (12.1) |  |
| *Thoracic* | 23 (0.4) | 7 (0.1) |  |
| *Neuro* | 1464 (25.2) | 1884 (28.5) |  |
| *Otolaryngology, Eye* | 917 (15.8) | 276 (4.2) |  |
| *Urology, gynecology* | 517 (8.9) | 234 (3.5) |  |
| Emergency | 1465 (25.3) | 1435 (21.7) | <0.001 |
| General anesthesia | 3803 (65.6) | 5829 (88.1) | <0.001 |
| ****Intraoperative variables*** |  |  |  |
| Operation duration, minute | 106.4 (±106.9) | 194.6 (±148.6) | <0.001 |
| Inotropic drug requirement | 1270 (21.9) | 3279 (49.6) | <0.001 |
| Red blood cell transfusion | 1286 (22.2) | 3198 (48.3) | <0.001 |
| ***Mortality*** |  |  |  |
| 30-day | 91 (1.6) | 251 (3.8) | <0.001 |
| In-hospital | 119 (2.1) | 347 (5.2) | <0.001 |

Data are presented as n (%) or mean (±standard deviation).

hs-cTn indicates high-sensitivity cardiac troponin; RAAS, renin-angiotensin-aldosterone system; ESC, European Society of Cardiology; ESA, European Society of Anaesthesiology.

**Supplemental Table 7. Baseline characteristics according to the diagnosis of myocardial injury after noncardiac surgery in the entire population before Propensity Score matching**

|  | LOD | | Mild elevation | |  |
| --- | --- | --- | --- | --- | --- |
|  | No diagnosed MINS (N=7314) | Diagnosed MINS (N=644) | No diagnosed MINS (N=3617) | Diagnosed MINS (N=840) | *P-*value |
| Preoperative hs-cTn I, ng/L | 6 | 6 | 16 (±8) | 20 (±10) | <0.001 |
| ***Preoperative variables*** |  |  |  |  |  |
| Male | 4020 (55.0) | 412 (64.0) | 2089 (57.8) | 514 (61.2) | <0.001 |
| Age | 57.8 (±18.1) | 60.3 (±15.7) | 63.0 (±17.2) | 68.0 (±14.8) | <0.001 |
| Hypertension | 3990 (54.6) | 427 (66.3) | 2629 (72.7) | 693 (82.5) | <0.001 |
| Diabetes | 1729 (23.6) | 195 (30.3) | 1185 (32.8) | 343 (40.8) | <0.001 |
| Coronary artery disease | 698 (9.5) | 104 (16.1) | 585 (16.2) | 226 (26.9) | <0.001 |
| Chronic kidney disease | 229 (3.1) | 39 (6.1) | 469 (13.0) | 161 (19.2) | <0.001 |
| Previous stroke | 432 (5.9) | 53 (8.2) | 297 (8.2) | 100 (11.9) | <0.001 |
| Arrhythmia | 294 (4.0) | 45 (7.0) | 341 (9.4) | 111 (13.2) | <0.001 |
| Current smoking | 612 (8.4) | 51 (7.9) | 247 (6.8) | 63 (7.5) | 0.04 |
| Preoperative hemoglobin, g/dl | 12.5 (±2.0) | 11.8 (±2.2) | 11.8 (±2.1) | 11.2 (±2.1) | <0.001 |
| Medication |  |  |  |  |  |
| Antiplatelet agent | 2037 (27.9) | 195 (30.3) | 1483 (41.0) | 438 (52.1) | <0.001 |
| Statin | 1987 (27.2) | 178 (27.6) | 1271 (35.1) | 366 (43.6) | <0.001 |
| Beta-blocker | 1477 (20.2) | 212 (32.9) | 1315 (36.4) | 395 (47.0) | <0.001 |
| Calcium channel blocker | 2359 (32.3) | 227 (35.2) | 1746 (48.3) | 434 (51.7) | <0.001 |
| RAAS inhibitor | 2042 (27.9) | 194 (30.1) | 1573 (43.5) | 428 (51.0) | <0.001 |
| ESC/ESA surgical risk |  |  |  |  | <0.001 |
| *Mild* | 2345 (32.1) | 96 (14.9) | 1036 (28.6) | 118 (14.0) |  |
| *Intermediate* | 4203 (57.5) | 351 (54.5) | 2272 (62.8) | 561 (66.8) |  |
| *High* | 766 (10.5) | 197 (30.6) | 309 (8.5) | 161 (19.2) |  |
| Operation type |  |  |  |  | <0.001 |
| *Vascular* | 860 (11.8) | 122 (18.9) | 501 (13.9) | 171 (20.4) |  |
| *Orthopedics* | 2065 (28.2) | 306 (47.5) | 1057 (29.2) | 321 (38.2) |  |
| *Abdominal* | 926 (12.7) | 58 (9.0) | 547 (15.1) | 159 (18.9) |  |
| *Thoracic* | 22 (0.3) | 0 | 8 (0.2) | 0 |  |
| *Neuro* | 2325 (31.8) | 112 (17.4) | 799 (22.1) | 112 (13.3) |  |
| *Otolaryngology, Eye* | 687 (9.4) | 20 (3.1) | 443 (12.2) | 43 (5.1) |  |
| *Urology, gynecology* | 429 (5.9) | 26 (4.0) | 262 (7.2) | 34 (4.0) |  |
| Emergency | 1523 (20.8) | 183 (28.4) | 893 (24.7) | 301 (35.8) | <0.001 |
| General anesthesia | 5882 (80.4) | 567 (88.0) | 2498 (69.1) | 685 (81.5) | <0.001 |
| ****Intraoperative variables*** |  |  |  |  |  |
| Operation duration, minute | 150.0 (±128.8) | 285.9 (±210.1) | 127.1 (±116.3) | 195.0 (±163.7) | <0.001 |
| Inotropic drug requirement | 2364 (32.3) | 434 (67.4) | 1241 (34.3) | 510 (60.7) | <0.001 |
| Red blood cell transfusion | 2732 (37.4) | 246 (38.2) | 1119 (30.9) | 387 (46.1) | <0.001 |

Data are presented as n (%) or mean (±standard deviation).

LOD indicates limit of detection; hs-cTn, high-sensitivity cardiac troponin; RAAS, renin-angiotensin-aldosterone system; ESC, European Society of Cardiology; ESA, European Society of Anaesthesiology.

**Supplemental Table 8. Baseline characteristics according to the diagnosis of myocardial injury after noncardiac surgery after Propensity Score matching**

|  | LOD | | Mild elevation | | *P*-value |
| --- | --- | --- | --- | --- | --- |
|  | No diagnosed MINS (N=3484) | Diagnosed MINS (N=385) | No diagnosed MINS (N=3185) | Diagnosed MINS  (N=684) |  |
| Preoperative hs-cTn I, ng/L | 6 | 6 | 15 (±8) | 19 (±10) |  |
| ***Preoperative variables*** |  |  |  |  |  |
| Male | 1955 (56.1) | 262 (68.1) | 1817 (57.0) | 416 (60.8) | <0.001 |
| Age | 62.6 (±16.0) | 63.0 (±15.7) | 62.2 (±17.6) | 66.8 (±15.4) | <0.001 |
| Hypertension | 2428 (69.7) | 294 (76.4) | 2202 (69.1) | 539 (78.8) | <0.001 |
| Diabetes | 1069 (30.7) | 127 (33.0) | 937 (29.4) | 253 (37.0) | 0.001 |
| Coronary artery disease | 501 (14.4) | 84 (21.8) | 415 (13.0) | 163 (23.8) | <0.001 |
| Chronic kidney disease | 225 (6.5) | 39 (10.1) | 197 (6.2) | 67 (9.8) | <0.001 |
| Previous stroke | 282 (8.1) | 39 (10.1) | 244 (7.7) | 69 (10.1) | 0.09 |
| Arrhythmia | 247 (7.1) | 40 (10.4) | 241 (7.6) | 62 (9.1) | 0.05 |
| Current smoking | 240 (6.9) | 31 (8.1) | 227 (7.1) | 57 (8.3) | 0.52 |
| Preoperative hemoglobin, g/dl | 12.0 (±2.0) | 11.5 (±2.1) | 12.0 (±2.1) | 11.4 (±2.1) | <0.001 |
| Medication |  |  |  |  |  |
| Antiplatelet agent | 1308 (37.5) | 145 (37.7) | 1155 (36.3) | 319 (46.6) | <0.001 |
| Statin | 1144 (32.8) | 119 (30.9) | 1006 (31.6) | 271 (39.6) | 0.001 |
| Beta-blocker | 1097 (31.5) | 165 (42.9) | 987 (31.0) | 279 (40.8) | <0.001 |
| Calcium channel blocker | 1554 (44.6) | 171 (44.4) | 1388 (43.6) | 315 (46.1) | 0.65 |
| RAAS inhibitor | 1376 (39.5) | 144 (37.4) | 1214 (38.1) | 306 (44.7) | 0.01 |
| ESC/ESA surgical risk |  |  |  |  | <0.001 |
| *Mild* | 1055 (30.3) | 57 (14.8) | 941 (29.5) | 101 (14.8) |  |
| *Intermediate* | 2046 (58.7) | 219 (56.9) | 1955 (61.4) | 442 (64.6) |  |
| *High* | 383 (11.0) | 109 (28.3) | 289 (9.1) | 141 (20.6) |  |
| Operation type |  |  |  |  | <0.001 |
| *Vascular* | 414 (11.9) | 75 (19.5) | 370 (11.6) | 137 (20.0) |  |
| *Orthopedics* | 1025 (29.4) | 173 (44.9) | 919 (28.9) | 273 (39.9) |  |
| *Abdominal* | 543 (15.6) | 47 (12.2) | 489 (15.4) | 107 (15.6) |  |
| *Thoracic* | 7 (0.2) | 0 | 8 (0.3) | 0 |  |
| *Neuro* | 946 (27.2) | 61 (15.8) | 775 (24.3) | 101 (14.8) |  |
| *Otolaryngology, Eye* | 330 (9.5) | 11 (2.9) | 392 (12.3) | 37 (5.4) |  |
| *Urology, gynecology* | 219 (6.3) | 18 (4.7) | 232 (7.3) | 29 (4.2) |  |
| Emergency | 872 (25.0) | 132 (34.3) | 770 (24.2) | 242 (35.4) | <0.001 |
| General anesthesia | 2575 (73.9) | 327 (84.9) | 2328 (73.1) | 577 (84.4) | <0.001 |
| ****Intraoperative variables*** |  |  |  |  |  |
| Operation duration, minute | 145.2 (±129.5) | 263.8 (±206.9) | 131.9 (±118.8) | 207.2 (±170.6) | <0.001 |
| Inotropic drug requirement | 1227 (35.2) | 260 (67.5) | 1094 (34.3) | 417 (61.0) | <0.001 |
| Red blood cell transfusion | 1288 (35.2) | 146 (37.9) | 1051 (33.0) | 318 (46.5) | <0.001 |

Data are presented as n (%) or mean (±standard deviation).

LOD indicates limit of detection; hs-cTn, high-sensitivity cardiac troponin; RAAS, renin-angiotensin-aldosterone system; ESC, European Society of Cardiology; ESA, European Society of Anaesthesiology

**Supplemental Table 9. Baseline characteristics according to the diagnosis of myocardial injury after noncardiac surgery in Patients with Postoperative hs-cTn before Propensity Score matching**

|  | LOD | | Mild elevation | |  |
| --- | --- | --- | --- | --- | --- |
|  | No diagnosed MINS (N=3442) | Diagnosed MINS (N=644) | No diagnosed MINS (N=1689) | Diagnosed MINS (N=840) | *P-*value |
| Preoperative hs-cTn I, ng/L | 6 | 6 | 16 (±8) | 20 (±10) |  |
| ***Preoperative variables*** |  |  |  |  |  |
| Male | 2051 (59.6) | 412 (64.0) | 1039 (61.5) | 514 (61.2) | 0.16 |
| Age | 62.1 (±14.2) | 60.3 (±15.7) | 65.8 (±14.4) | 68.0 (±14.8) | <0.001 |
| Hypertension | 2182 (63.4 | 427 (66.3) | 1293 (76.6) | 693 (82.5) | <0.001 |
| Diabetes | 963 (28.0) | 195 (30.3) | 575 (34.0) | 343 (40.8) | <0.001 |
| Coronary artery disease | 478 (13.9) | 104 (16.1) | 339 (20.1) | 226 (26.9) | <0.001 |
| Chronic kidney disease | 106 (3.1) | 39 (6.1) | 174 (10.3) | 161 (19.2) | <0.001 |
| Previous stroke | 257 (7.5) | 53 (8.2) | 149 (8.8) | 100 (11.9) | <0.001 |
| Arrhythmia | 179 (5.2) | 45 (7.0) | 189 (11.2) | 111 (13.2) | <0.001 |
| Current smoking | 331 (9.6) | 51 (7.9) | 138 (8.2) | 63 (7.5) | 0.11 |
| Preoperative hemoglobin, g/dl | 12.6 (±2.0) | 11.8 (±2.2) | 12.0 (±2.1) | 11.2 (±2.1) | <0.001 |
| Medication |  |  |  |  |  |
| Antiplatelet agent | 1232 (35.8) | 195 (30.3) | 776 (45.9) | 438 (52.1) | <0.001 |
| Statin | 1165 (33.8) | 178 (27.6) | 670 (39.7) | 366 (43.6) | <0.001 |
| Beta-blocker | 841 (24.4) | 212 (32.9) | 666 (39.4) | 395 (47.0) | <0.001 |
| Calcium channel blocker | 1235 (35.9) | 227 (35.2) | 828 (49.0) | 434 (51.7) | <0.001 |
| RAAS inhibitor | 1156 (33.6) | 194 (30.1) | 788 (46.7) | 428 (51.0) | <0.001 |
| ESC/ESA surgical risk |  |  |  |  | <0.001 |
| *Mild* | 784 (22.8) | 96 (14.9) | 328 (19.4) | 118 (14.0) |  |
| *Intermediate* | 2011 (58.4) | 351 (54.5) | 1111 (65.8) | 561 (66.8) |  |
| *High* | 647 (18.8) | 197 (30.6) | 250 (14.8) | 161 (19.2) |  |
| Operation type |  |  |  |  | <0.001 |
| *Vascular* | 629 (18.3) | 122 (18.9) | 285 (16.9) | 171 (20.4) |  |
| *Orthopedics* | 1010 (29.3) | 306 (47.5) | 571 (33.8) | 321 (38.2) |  |
| *Abdominal* | 319 (9.3) | 58 (9.0) | 263 (15.6) | 159 (18.9) |  |
| *Thoracic* | 5 (0.1) | 0 | 2 (0.1) | 0 |  |
| *Neuro* | 1256 (36.5) | 112 (17.4) | 404 (23.9) | 112 (13.3) |  |
| *Otolaryngology, Eye* | 120 (3.5) | 20 (3.1) | 93 (5.5) | 43 (5.1) |  |
| *Urology, gynecology* | 103 (3.0) | 26 (4.0) | 71 (4.2) | 34 (4.0) |  |
| Emergency | 574 (16.7) | 183 (28.4) | 377 (22.3) | 301 (35.8) | <0.001 |
| General anesthesia | 3146 (91.4) | 567 (88.0) | 1431 (84.7) | 685 (81.5) | <0.001 |
| ****Intraoperative variables*** |  |  |  |  |  |
| Operation duration, minute | 192.7 (±135.9) | 285.9 (±210.1) | 163.6 (±120.9) | 195.0 (±163.7) | <0.001 |
| Inotropic drug requirement | 1551 (45.1) | 434 (67.4) | 784 (46.4) | 510 (60.7) | <0.001 |
| Red blood cell transfusion | 1807 (52.5) | 246 (38.2) | 785 (44.9) | 387 (46.1) | <0.001 |

Data are presented as n (%) or mean (±standard deviation).

LOD indicates limit of detection; hs-cTn, high-sensitivity cardiac troponin; RAAS, renin-angiotensin-aldosterone system; ESC, European Society of Cardiology; ESA, European Society of Anaesthesiology.

**Supplemental Table 10. Baseline Characteristics According to the Diagnosis of Myocardial Injury After Noncardiac Surgery in Patients with Postoperative hs-cTn after Propensity Score matching**

|  | LOD | | Mild elevation | | *P*-value |
| --- | --- | --- | --- | --- | --- |
|  | No diagnosed MINS (N=1757) | Diagnosed MINS (N=385) | No diagnosed MINS (N=1527) | Diagnosed MINS  (N=684) |  |
| Preoperative hs-cTn I, ng/L | 6 | 6 | 15 (±8) | 19 (±10) |  |
| ***Preoperative variables*** |  |  |  |  |  |
| Male | 1050 (59.8) | 262 (68.1) | 934 (61.2) | 416 (60.8) | 0.03 |
| Age | 65.4 (±13.0) | 63.0 (±15.7) | 65.3 (±14.6) | 66.8 (±15.4) | <0.001 |
| Hypertension | 1330 (75.7) | 294 (76.4) | 1134 (74.3) | 539 (78.8) | 0.15 |
| Diabetes | 601 (34.2) | 127 (33.0) | 487 (31.9) | 253 (37.0) | 0.12 |
| Coronary artery disease | 348 (19.8) | 84 (21.8) | 267 (17.5) | 163 (23.8) | 0.004 |
| Chronic kidney disease | 105 (6.0) | 39 (10.1) | 92 (6.0) | 67 (9.8) | <0.001 |
| Previous stroke | 172 (9.8) | 39 (10.1) | 131 (8.6) | 69 (10.1) | 0.55 |
| Arrhythmia | 153 (8.7) | 40 (10.4) | 139 (9.1) | 62 (9.1) | 0.78 |
| Current smoking | 142 (6.9) | 31 (8.1) | 227 (7.1) | 57 (8.3) | 0.52 |
| Preoperative hemoglobin, g/dl | 12.1 (±2.0) | 11.5 (±2.1) | 12.2 (±2.1) | 11.4 (±2.1) | <0.001 |
| Medication |  |  |  |  |  |
| Antiplatelet agent | 775 (43.5) | 145 (37.7) | 650 (42.6) | 319 (46.6) | 0.03 |
| Statin | 670 (38.1) | 119 (30.9) | 572 (37.5) | 271 (39.6) | 0.03 |
| Beta-blocker | 639 (36.4) | 165 (42.9) | 541 (35.4) | 279 (40.8) | 0.01 |
| Calcium channel blocker | 815 (46.4) | 171 (44.4) | 706 (46.2) | 315 (46.1) | 0.92 |
| RAAS inhibitor | 764 (43.5) | 144 (37.4) | 657 (43.0) | 306 (44.7) | 0.12 |
| ESC/ESA surgical risk |  |  |  |  | <0.001 |
| *Mild* | 374 (21.3) | 57 (14.8) | 304 (19.9) | 101 (14.8) |  |
| *Intermediate* | 1048 (59.6) | 219 (56.9) | 990 (64.8) | 442 (64.6) |  |
| *High* | 335 (19.1) | 109 (28.3) | 233 (15.3) | 141 (20.6) |  |
| Operation type |  |  |  |  | <0.001 |
| *Vascular* | 314 (17.9) | 75 (19.5) | 251 (16.4) | 137 (20.0) |  |
| *Orthopedics* | 582 (33.1) | 173 (44.9) | 503 (32.9) | 273 (39.9) |  |
| *Abdominal* | 219 (12.5) | 47 (12.2) | 227 (14.9) | 107 (15.6) |  |
| *Thoracic* | 3 (0.2) | 0 | 2 (0.1) | 0 |  |
| *Neuro* | 509 (29.0) | 61 (15.8) | 394 (25.8) | 101 (14.8) |  |
| *Otolaryngology, Eye* | 68 (3.9) | 11 (2.9) | 87 (5.7) | 37 (5.4) |  |
| *Urology, gynecology* | 62 (3.5) | 18 (4.7) | 63 (4.1) | 29 (4.2) |  |
| Emergency | 872 (25.0) | 132 (34.3) | 770 (24.2) | 242 (35.4) | <0.001 |
| General anesthesia | 2575 (73.9) | 327 (84.9) | 2328 (73.1) | 577 (84.4) | <0.001 |
| ****Intraoperative variables*** |  |  |  |  |  |
| Operation duration, minute | 185.2 (±137.6) | 263.8 (±206.9) | 167.3 (±122.0) | 207.2 (±170.6) | <0.001 |
| Inotropic drug requirement | 842 (47.9) | 260 (67.5) | 693 (45.4) | 417 (61.0) | <0.001 |
| Red blood cell transfusion | 846 (48.2) | 146 (37.9) | 716 (46.9) | 318 (46.5) | 0.004 |

Data are presented as n (%) or mean (±standard deviation).

LOD indicates limit of detection; hs-cTn, high-sensitivity cardiac troponin; RAAS, renin-angiotensin-aldosterone system; ESC, European Society of Cardiology; ESA, European Society of Anaesthesiology.
